# Response Gene to Complement 32 in Vascular Diseases

**DOI:** 10.3389/fcvm.2018.00128

**Published:** 2018-09-18

**Authors:** Xiao-Bing Cui, Shi-You Chen

**Affiliations:** Department of Physiology & Pharmacology, University of Georgia, Athens, GA, United States

**Keywords:** response gene to complement 32, vascular diseases, smooth muscle cells, endothelial cells, macrophages, T-lymphocyte cells

## Abstract

Response gene to complement 32 (RGC32) is a protein that was identified in rat oligodendrocytes after complement activation. It is expressed in most of the organs and tissues, such as brain, placenta, heart, and the liver. Functionally, RGC32 is involved in various physiological and pathological processes, including cell proliferation, differentiation, fibrosis, metabolic disease, and cancer. Emerging evidences support the roles of RGC32 in vascular diseases. RGC32 promotes injury-induced vascular neointima formation by mediating smooth muscle cell (SMC) proliferation and migration. Moreover, RGC32 mediates endothelial cell activation and facilitates atherosclerosis development. Its involvement in macrophage phagocytosis and activation as well as T-lymphocyte cell cycle activation also suggests that RGC32 is important for the development and progression of inflammatory vascular diseases. In this mini-review, we provide an overview on the roles of RGC32 in regulating functions of SMCs, endothelial cells, and immune cells, and discuss their contributions to vascular diseases.

## Introduction

Response gene to complement 32 (RGC32) was first cloned from rat oligodendrocytes by differential display screening for genes responding to complement activation (1). Oligodendrocytes are the targets of immune-mediated attack in experimental allergic encephalomyelitis and multiple sclerosis. Sublytic complement attack on oligodendrocytes induces changes in cellular phenotype, which are potentially beneficial to the cell. However, molecular mechanisms underlying these effects are poorly understood. To screen for genes expressed by oligodendrocytes in response to complement activation, Badea et al. treated primary rat oligodendrocytes with anti-galactocerebroside antibody and normal human serum, which was used as a source of complement (1). Through differential display PCR, they identified 32 genes with altered mRNA expression in response to the complement activation. These genes were designated as *RGC1* to *32* according to the order of identification. The rat *Rgc32* encodes a 14.7 kDa protein with 137 amino acids without homology to any other proteins and contains no motif of known biochemical function. In addition, RGC32 does not have signal sequences, and hydrophobicity analysis finds no transmembrane domains (1). Therefore, *Rgc32* represents a prototype of a novel type of gene. The human RGC32 gene is located on chromosome 13 and encodes a 137-amino-acid protein with 92% similarity to rat and mouse RGC32 (2). RGC32 is expressed in numerous organs and tissues including placenta, kidney, brain, liver, heart, adipose tissue, smooth muscle cells (SMCs), endothelial cells (ECs), and macrophages (2–6). Functionally, it is involved in various physiological and pathological processes, such as cell proliferation, differentiation, epithelial–mesenchymal transition, and fibroblast activation (2–4, 7, 8). RGC32 has been found to physically associate with cyclin-dependent kinase p34^CDC2^ and enhances its kinase activity to induce quiescent aortic SMCs to enter cell cycle S phase, indicating that RGC32 promotes SMC proliferation (2). Surprisingly, RGC32 causes G2/M arrest in glioma by forming a protein complex with polo-like kinase 1 (9). These results suggest that depending on cell type and cellular environments RGC32 could either promote or block cell cycle progression. In addition to cell proliferation, RGC32 regulates SMC differentiation (3) and epithelial-mesenchymal transition of human renal proximal tubular cells by interacting with Smad3 (7, 10). Moreover, RGC32 contributes to the development of several other diseases, such as cancer (11, 12), multiple sclerosis (13), and metabolic disorders (5, 6). In this mini-review, we will focus on the roles of RGC32 in the development of vascular diseases, particularly its roles in regulating the functional properties of SMCs, ECs, and immune cells.

## Roles of RGC32 in SMCs

Vascular SMCs form the muscular layer of blood vessel walls and regulate arterial tone and blood pressure. These cells possess a remarkable plasticity allowing mature contractile SMCs to dedifferentiate, which enables vessel growth and repair. SMC phenotypic modulation contributes to multiple cardiovascular pathologies, including atherosclerosis, aneurysm, pulmonary hypertension, transplant vasculopathy, and hypertension (14). RGC32 regulates different aspects of SMC phenotype via distinct mechanisms in a context-dependent manner. First, RGC32 mediates transforming growth factor β (TGF-β)-induced SMC differentiation (3, 15). TGF-β signaling plays pivotal roles in SMC differentiation during vascular development as well as phenotypic switching in disease states. TGF-β elicits its effects through specific type I and type II serine/threonine kinase receptors and intracellular Smad transcription factors. TGF-β induces RGC32 mRNA expression through activating and recruiting Smad2, Smad4, and polyomavirus enhancer activator (PEA3) to the RGC32 promoter in Monc-1 neural crest cells (15). Knockdown of RGC32 inhibits, while overexpression of RGC32 enhances, TGF-β-induced SMC marker gene expression. RGC32 mediates SMC differentiation in a CArG-dependent manner (3). Second, RGC32 regulates SMC proliferation. Sublytic complement activation enhances RGC32 mRNA expression in human aortic SMCs and induces its nuclear translocation. RGC32 is phosphorylated by cyclin-dependent kinase p34^CDC2^-cyclin B1 at Thr-91, physically interacts with p34^CDC2^ to increase its kinase activity, and induces quiescent aortic SMCs to enter S-phase (2). In rat aortic SMCs, RGC32 also promotes p34^CDC2^ (Thr161) phosphorylation and induces SMC proliferation (16). Third, RGC32 stimulates SMC migration through induction of focal adhesion contact and stress fiber formation. These effects are caused by the enhanced rho kinase II-α activity due to RGC32-induced downregulation of Rad GTPase expression (16). Overexpression of RGC32 blocks, whereas knockdown of RGC32 increases Rad GTPase expression. *In vivo*, RGC32 expression is increased along with the progression of intimal hyperplasia in a rat carotid artery balloon-injury model. Knockdown of RGC32 using short hairpin RNA via adenovirus-mediated gene delivery inhibits, while RGC32 overexpression promotes neointima formation in injured rat carotid artery (16). Elevated RGC32 expression is also found in SMCs of human atherosclerotic lesions (17), suggesting that RGC32 may contribute to the development of atherosclerosis.

## Roles of RGC32 in ECs

The ECs constitute a single layer lining the blood luminal surface of vessels. These cells exhibit distinct and unique functions vital to vascular biology, including blood vessel tone, hemostasis, neutrophil recruitment, and hormone trafficking. EC dysfunction, characterized by its reduced vasodilation, proinflammatory state, and prothrombic properties, is associated with various cardiovascular diseases, including hypertension, coronary artery disease, chronic heart failure, peripheral vascular disease, and diabetes (18). RGC32 appears to be important for EC proliferation and angiogenesis. Hypoxia, an angiogenesis stimulator, induces RGC32 expression in human umbilical vein ECs (HUVECs) (19). Hypoxia induces RGC32 expression at both transcriptional and posttranscriptional levels. Hypoxia increases RGC32 mRNA expression by promoting hypoxia-inducible factor-1α (HIF-1α) binding to RGC32 promoter and thus increasing its promoter activity. Hypoxia also prolongs RGC32 mRNA half-life that accounts for its elevated protein level (19). Functionally, overexpression of RGC32 reduces the proliferation and migration of HUVECs, destabilizes vascular structure formation *in vitro*, and inhibits vascular endothelial growth factor (VEGF)-induced angiogenesis in Matrigel assays *in vivo* (19). RGC32 overexpression also decreases the recovery of blood flow in mouse hind-limb ischemia model, and suppresses tumor growth that is associated with the reduced angiogenesis (19). Moreover, RGC32 impedes EC proliferation by inhibiting the expression of fibroblast growth factor 2 (FGF2) and cyclin E without affecting VEGF downstream pathways (19).

It appears that RGC32 regulates the growth of human aortic ECs differently from HUVECs. Instead of blocking the proliferation, RGC32 promotes C5b-9- and serum growth factors-induced proliferation and CDC2 activation. RGC32 physically associates with Akt to increase Akt phosphorylation at Ser473, which further activates CDC2 (4). In addition, RGC32 also mediates human aortic EC proliferation indirectly by promoting the release of growth factors from ECs. Knockdown of RGC32 increases the expression and release of C-X-C motif chemokine 5 (CXCL5), interleukin (IL)-8, tissue inhibitor of metalloproteinases 1 (TIMP1), and VEGF-D while decreases the production of leptin, placenta growth factor (PlGF), and regulated on activation normal T cell expressed and secreted (RANTES) in human aortic ECs (4).

Our study demonstrates that absence of RGC32 in mice causes fetal growth restriction through interrupting the placental angiogenesis, which is due to the decreased VEGF receptor 2 expression in ECs and PlGF expression in trophoblasts (20). RGC32-deficient (*Rgc32*^−/−^) embryos and fetal placentas at 16.5 days post-coitum are significantly smaller than the wild-type and exhibit defective angiogenesis, which causes smaller body sizes when they are born (20). PCR array shows that VEGF receptor 2 and PlGF are down-regulated in *Rgc32*^−/−^ placentas. Mechanistically, RGC32 increases VEGF receptor 2 expression through activating nuclear factor (NF)-κB signaling pathway (20). Reduced RGC32 expression is also found in human preeclamptic placentas compared with normal controls (21), indicating the important role of RGC32 in placenta functions. These studies suggest that RGC32 may exert different roles in EC proliferation and angiogenesis in different physiological or pathological processes, which likely depends on the location of the ECs or the nature of the stimulus.

A predominantly elevated RGC32 expression is observed in ECs in atherosclerotic lesions from both human and mouse (22). *Rgc32*^−/−^ attenuates both diet-induced and spontaneously developed atherosclerotic lesions in apolipoprotein E deficient (*Apoe*^−/−^) mice. Transplantation with wild-type bone marrow to *Rgc32*^−/−^ mice does not alter the protective effects of *Rgc32* deletion on atherosclerosis development, suggesting the critical roles of resident vascular cell RGC32 in the lesion development. Of importance, *Rgc32*^−/−^ decreases the macrophage content in the lesions without altering collagen and SMC contents. *In vitro*, RGC32 promotes tumor necrosis factor (TNF)-α-induced monocyte-EC interaction by upregulating intercellular adhesion molecule (ICAM)-1 and vascular cell adhesion molecule (VCAM)-1 expression in ECs. Interestingly, *Rgc32*^−/−^ has no effect on lesional macrophage proliferation (22). Vlaicu et al.'s study also shows an increased RGC32 expression in human atherosclerotic lesions. Knockdown of RGC32 blocks C5b-9-induced human aortic EC proliferation and migration (17). Both studies suggest an important role of RGC32 in EC activation and atherosclerosis development.

EC dysfunction is known to contribute to the pathogenesis of diabetes, insulin resistance and obesity (23, 24). Glucose and insulin induce RGC32 expression in human microvascular ECs *in vitro* through phosphatidylinositol 3-kinase (PI3K)-Akt pathway (25). Overexpression of RGC32 decreases the expression of glutamine-fructose-6-phosphate aminotransferase [isomerizing] 1 (GFPT1) and solute carrier family 2 member 12 (GLUT12), but increases glucagon-like peptide 2 receptor (GLP2R) expression in ECs. EC specific RGC32 overexpression improves the ability of glucose disposal without affecting the insulin sensitivity of the mice under high-fat diet conditions (25), suggesting RGC32 protects EC from high-fat diet-induced dysfunction. Our studies demonstrate that RGC32 contributes to high-fat diet-induced metabolic dyshomeostasis by increasing adipose tissue inflammation and facilitating hepatic lipogenesis (5, 6), indicating RGC32 functions differently in different cell, tissue or organs even in the same physiological or pathological condition.

## Roles of RGC32 in immune cells

Immune system activation or dysregulation plays important roles in the development and progression of numerous vascular diseases (26, 27). RGC32 has been found to regulate the functions of different immune cells. RGC32 level is higher in unstimulated peripheral CD14^+^ monocytes of patients with hyper-immunoglobulin E syndrome compared with healthy controls (28). RGC32 is also expressed in CD3^+^ and CD68^+^ cells in brains of multiple sclerosis patients as well as in peripheral blood CD4^+^ cells. Its expression in peripheral blood CD4^+^ cells is elevated in stable multiple sclerosis patients as compared to healthy subjects or patients with relapses (13). Functionally, RGC32 acts as a negative cell cycle regulator in T-lymphocytes (29). *Rgc32*^−/−^ causes an increased proliferation of both CD4^+^ and CD8^+^ T cells. The augmented T-lymphocyte proliferation may be due to an increased IL-2 expression, which is regulated by PI3K signaling (29).

In addition to T cells, RGC32 also regulates macrophage function. Although RGC32 is induced during monocyte-macrophage differentiation, RGC32 is not important for this process because *Rgc32*^−/−^ bone marrow progenitor cells can differentiate normally to macrophages (30). However, RGC32 is essential for macrophage phagocytosis. Peritoneal and bone marrow-derived macrophages with *Rgc32* deletion exhibit significant defects in phagocytosis (30). Conversely, RGC32 overexpression increases the phagocytosis (30). Mechanistically, RGC32 is recruited to macrophage membrane and directly binds to protein kinase C, which induces F-actin assembly and the formation of phagocytic cups (30).

RGC32 is also involved in classical and alternative macrophage activation (31, 32). An *in vitro* study using THP-1 cells indicates that RGC32 expression is induced by IL-4, while inhibited by lipopolysaccharides (LPS). Tumor-associated macrophages, which are considered as the alternatively activated macrophage phenotype, express high levels of RGC32 (31), suggesting that RGC32 may be related to the function of alternatively activated macrophages. Indeed, RGC32 suppresses the production of pro-inflammatory cytokine IL-6, while promotes the production of anti-inflammatory mediator TGF-β. However, our studies show that interferon-γ and LPS induce RGC32 expression in mouse primary peritoneal and bone marrow-derived macrophages (32). Deletion of *Rgc32* impairs the classic macrophage activation and decreases the production of inducible nitric oxide synthase (iNOS) and IL-1β through blocking NF-κB binding to their promoters. *In vivo, Rgc32*^−/−^ in mice improves bleomycin-induced skin and lung sclerosis by impeding macrophage accumulation and inhibiting the expression of iNOS and IL-1β in macrophages (32). The discrepancy among the results from THP-1 cells and the primary mouse monocytes/macrophages may be due to the different intrinsic cell properties and the stimulators used in the assay. Nevertheless, these results highlight the roles of RGC32 in both classical and alternative macrophage activation.

## Summary and perspective

**Figure 1:**
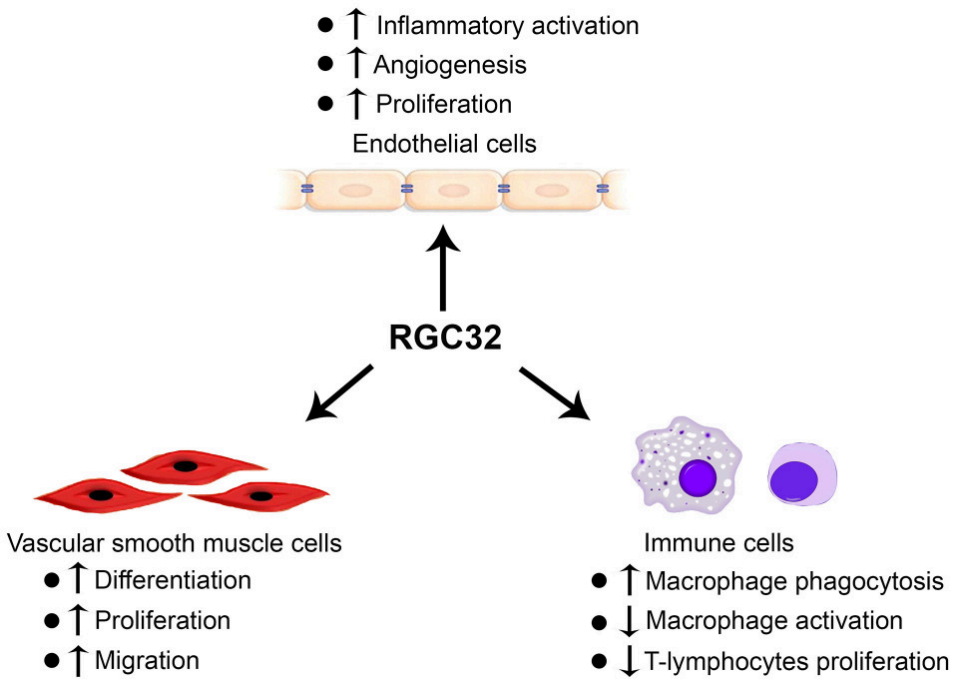
RGC32 functions in endothelial cells, vascular smooth muscle cells, and immune cells.

Tremendous efforts from several different laboratories have been made to establish the important roles of RGC32 in SMCs, ECs, and immune cells (Figure 1). However, the mechanisms underlying RGC32 functions remain largely unknown. Therefore, extensive future studies are warranted. For example, although RGC32 function in the development of atherosclerosis is established, further investigation is needed in order to elucidate the effects of RGC32 on other vascular diseases, such as aortic aneurysm, hypertension, and thrombosis. In addition, tissue-specific *Rgc32* deficient and/or transgenic mouse models are likely to be the essential and valuable tools to dissect the specific functions of RGC32 in SMCs, ECs, macrophages, T cells, and/or other immune cells in the development and/or progression of different vascular diseases.

## Author contributions

All authors listed have made a substantial, direct and intellectual contribution to the work, and approved it for publication.

### Conflict of interest statement

The authors declare that the research was conducted in the absence of any commercial or financial relationships that could be construed as a potential conflict of interest.
